# Distributed multi-robot LiDAR SLAM with ground-optimized preprocessing and slope-adaptive segmentation

**DOI:** 10.3389/fnbot.2026.1839252

**Published:** 2026-07-01

**Authors:** Yu Wang, Qiang Zou, Fei Wang

**Affiliations:** Faculty of Robot Science and Engineering, Northeastern University, Shenyang, China

**Keywords:** distributed multi-robot SLAM, field robotics, ground segmentation, IMU-guided parameter tuning, LiDAR odometry, slope adaptation

## Abstract

Large-scale outdoor robot navigation increasingly demands SLAM systems capable of operating efficiently across diverse and challenging terrain. While single-robot approaches face inherent coverage and computational limitations, distributed multi-robot frameworks extend this capability through collaborative mapping—yet they still degrade in complex outdoor environments due to two unresolved challenges: redundant ground points in raw point clouds overload feature extraction and loop-closure matching, while fixed ground segmentation thresholds fail on sloped terrain causing misclassification and trajectory degradation. We address the first challenge by integrating ground segmentation preprocessing as a parallel stage for each robot within the distributed SLAM framework, reducing point cloud size by 50.78% and achieving a 21.4% RMSE improvement for Robot 0 (7.99 m → 6.28 m) compared to the unprocessed baseline. We address the second challenge with the proposed SAGS (Slope-Adaptive Ground Segmentation) module, which continuously monitors platform tilt via IMU orientation and dynamically interpolates ground segmentation parameters within a 5°–15° tilt range; SAGS recovers Robot 1 RMSE from 8.48 m to 6.23 m (26.5% improvement) on sloped terrain without flat-terrain penalty (GPS-validated 1.083 m RMSE on a public 612 m benchmark). Both contributions are validated through progressive three-stage ablation evaluation on a campus three-robot dataset (heterogeneous team: two wheeled ground robots and one legged quadruped, diverse terrain including sloped sections) and cross-validated on a public GPS benchmark (612 m, GPS ground truth), confirming the independent contribution of each system component.

## 1 Introduction

Simultaneous Localization and Mapping (SLAM) is fundamental for autonomous navigation in large-scale, GPS-denied outdoor environments. Single-robot systems face inherent limitations in coverage, fault tolerance, and computational scalability when deployed over extensive areas (Saeedi et al., 2016). Multi-robot SLAM extends this capability by distributing exploration and computation across a robot team, enabling collaborative mapping with enhanced efficiency and resilience. Distributed architectures achieve scalability through peer-to-peer communication and two-stage global-local pose graph optimization, allowing each robot to solve local optimization independently while periodically coordinating global consistency. However, distributed LiDAR SLAM still degrades on complex outdoor terrain due to two unresolved problems: (1) raw point clouds contain approximately 50% ground points that burden feature extraction and loop-closure matching with geometrically repetitive, low-discriminative information; and (2) ground segmentation methods calibrated for flat terrain fail when robots traverse slopes, causing misclassification and trajectory degradation.

Ground segmentation addresses the first problem by removing repetitive ground planes, reducing computational load and improving feature quality. State-of-the-art approaches achieve robust performance across varied terrain by estimating thresholds adaptively from the statistical distribution of identified ground points. Systems exploiting such ground-optimized preprocessing have demonstrated consistent improvements in both odometry accuracy and computational efficiency for single-robot platforms. However, this preprocessing strategy has not been applied to distributed multi-robot SLAM frameworks, leaving its efficiency and accuracy benefits unrealized in collaborative settings. Furthermore, all existing approaches rely on static segmentation thresholds calibrated for flat terrain—directly exposing the second problem: when a robot traverses a slope, platform tilt causes genuine ground points to violate the uprightness and distance criteria and be incorrectly labeled as obstacles, corrupting feature matching and degrading trajectory estimation.

This slope-induced failure manifests concretely in our experiments: Robot 1, traversing a sloped section of our campus testbed, degrades from 6.27 m RMSE without preprocessing to 8.48 m RMSE when fixed ground segmentation is applied—a 35.2% accuracy regression. The mis-segmented ground points inflate the apparent obstacle density and ultimately degrade trajectory estimation. No existing work addresses slope-induced ground segmentation failure in distributed multi-robot SLAM systems.

This paper integrates three components into a unified distributed multi-robot LiDAR SLAM system: a distributed SLAM backend for scalable pose graph optimization, ground segmentation preprocessing deployed in parallel per robot, and the proposed SAGS (Slope-Adaptive Ground Segmentation) module for slope-adaptive parameter control. The first two components together resolve the ground-point redundancy problem (contribution 1); SAGS then resolves slope-induced segmentation failure (contribution 2), restoring accuracy on sloped terrain without sacrificing flat-terrain performance.

The main contributions of this work are:

- We integrate ground segmentation preprocessing as a parallel stage into the distributed multi-robot LiDAR SLAM framework for the first time, achieving 50.78% point cloud reduction and 21.4% RMSE improvement for Robot 0 (7.99 m → 6.28 m) compared to the baseline without preprocessing.
- We propose SAGS, a slope-adaptive ground segmentation module that dynamically adjusts uprightness and distance thresholds based on real-time IMU tilt estimation, recovering trajectory accuracy on sloped terrain (Robot 1: 8.48 m → 6.23 m RMSE) while introducing no degradation on flat terrain.
- We conduct comprehensive evaluation including a progressive three-stage ablation study (C0: baseline, C1: +ground preprocessing, C2: +SAGS) that isolates each contribution's effect on both flat and slope terrain, plus cross-dataset validation on a public GPS benchmark (612 m), confirming zero flat-terrain penalty and cross-platform generalization.

## 2 Related work

### 2.1 Distributed multi-robot SLAM architectures

Multi-robot SLAM architectures can be categorized by their computational topology into centralized, decentralized, and distributed approaches. Centralized systems aggregate all sensor data on a central server for global optimization, achieving globally consistent maps at the cost of communication bottlenecks and single-point failure vulnerabilities.

Distributed architectures balance computational load through peer-to-peer communication and local optimization. The fundamental principle is to decompose the global multi-robot SLAM objective into intra-robot constraints (comprising local odometry factors) and inter-robot constraints (from loop closures between robots), enabling each robot to solve its local optimization independently with periodic global coordination. Recent implementations incorporate semantic information for robust data association (Chang et al., 2021), outlier-resilient optimization for handling erroneous loop closures (Lajoie et al., 2020), and sparse decentralized collaboration protocols (Lajoie and Beltrame, 2024).

For LiDAR-based systems, a two-stage global-local optimization framework effectively addresses coordinate frame alignment: first optimizing inter-robot transformation matrices **T**_βα_∈*SE*(3), then performing local pose graph optimization with transformed constraints. This approach reduces communication overhead while maintaining mapping accuracy comparable to centralized methods.

### 2.2 Ground segmentation methodologies

Ground segmentation separates horizontal supporting surfaces from vertical obstacles in 3D point clouds, providing multiple benefits: reduced computational complexity, improved obstacle detection, and enhanced motion estimation accuracy through feature quality improvement.

Ground segmentation approaches can be categorized by their underlying principles: (1) learning-based methods (Paigwar et al., 2020; Qi et al., 2017) that train neural networks to classify ground vs. non-ground points from labeled data, (2) elevation-based methods (Steinke et al., 2024) that threshold points based on height above an estimated ground plane, and (3) geometric methods that combine plane fitting with statistical analysis for adaptive parameter estimation.

Fixed-threshold methods fail in diverse terrain conditions. Adaptive approaches such as Patchwork++ (Lee et al., 2022) estimate elevation and flatness tolerance from the statistical distribution of definitively identified ground points, computing confidence-weighted thresholds that adjust proportionally to observed terrain statistics. This data-driven mechanism enables robust segmentation across flat roads, rough ground, and variable slopes without requiring manual tuning. Systems employing dual-threshold filtering (Pan et al., 2021) and ground constraints for indoor environments (Wei et al., 2022) demonstrate the effectiveness of ground-aware processing for odometry estimation. Place recognition descriptors computed from ground-removed point clouds (Kim and Kim, 2018) also benefit, as vertical structures possess higher spatial uniqueness than ground planes.

Despite advances in single-robot ground-optimized SLAM, no existing work addresses slope-induced ground segmentation failure in distributed multi-robot systems. When a robot traverses a ramp, platform tilt causes fixed Patchwork++ uprightness and distance thresholds to misclassify genuine ground points as obstacles. SAGS fills this gap by coupling IMU tilt estimation to real-time parameter adaptation.

## 3 Materials and methods

### 3.1 System overview

We formulate multi-robot SLAM as a nonlinear least squares optimization over robot pose sequences. For a team of *n* robots N={1,2,…,n}, the objective function aggregates both intra-robot odometry constraints $C_{\alpha }$ and inter-robot loop closure constraints $C_{\alpha \beta }$ in Equation 1:


$$\begin{array}{l} {\text{X}}^{*}=\arg\underset{\text{X}}{\min}\left\{\underset{\alpha \in N}{\sum }\underset{\left(i,j\right)\in C_{\alpha }}{\sum }{\text{F}}_{ij}+\underset{\begin{array}{c} \alpha ,\beta \in N\\ \alpha \neq \beta \end{array}}{\sum }\underset{\left(i,j\right)\in C_{\alpha \beta }}{\sum }{\text{F}}_{ij}\right\} \end{array}$$ (1)

where each constraint's cost function measures the discrepancy between observed and expected relative transformations using the standard graph-SLAM formulation (Grisetti et al., 2010).

The proposed system makes two novel contributions to the distributed multi-robot SLAM pipeline: (1) parallel Patchwork++ ground segmentation for each robot, and (2) the SAGS module that dynamically adapts segmentation parameters to terrain tilt.

**Figure 1:**
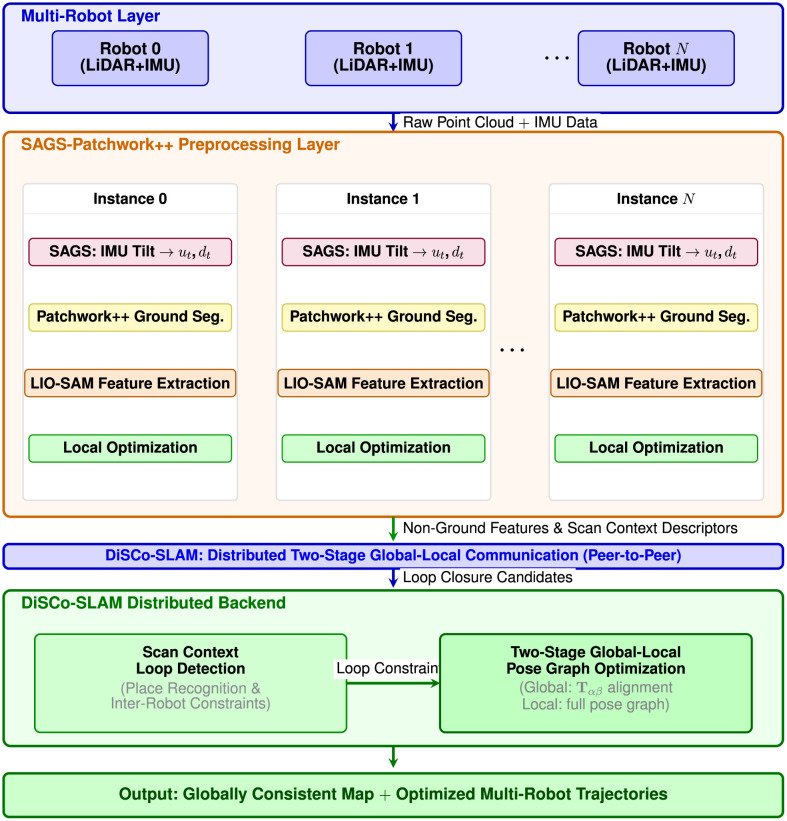
System architecture: Patchwork++ ground segmentation instances (with SAGS adaptive control) are deployed in parallel for each robot and integrated with the DiSCo-SLAM distributed backend for multi-robot pose graph optimization. Raw point clouds from multiple robots are first processed by parallel SAGS-controlled Patchwork++ instances, producing non-ground point clouds that feed the distributed SLAM backend for local odometry, inter-robot loop detection, and two-stage pose graph optimization.

As illustrated in Figure 1, the proposed system consists of two main processing stages: (1) parallel SAGS-controlled Patchwork++ ground segmentation and (2) DiSCo-SLAM distributed backend optimization. Each robot operates an independent ground segmentation instance that partitions incoming point clouds into ground and non-ground subsets. The non-ground point cloud $P_{ng}$ is subsequently processed for feature extraction, local odometry estimation, and inter-robot place recognition. This architecture achieves: (1) computational load reduction through point cloud size minimization ($\left|P_{ng}|\approx 0.5|P\right|$), reducing feature extraction complexity O(|P|) and ICP registration complexity O(|P|log|P|) (with KD-tree acceleration) proportionally; (2) feature quality improvement by removing geometrically repetitive ground planes—ground planes exhibit high geometric similarity across locations and can cause false positive inter-robot loop closures, while descriptors computed from $P_{ng}$ encode only vertical structures (buildings, trees, poles) with higher spatial uniqueness; and (3) parallel scalability as each robot's segmentation operates independently without inter-process communication.

### 3.2 Patchwork++ ground segmentation

Integrating ground segmentation preprocessing into the distributed multi-robot pipeline constitutes the first contribution of this work.

Patchwork++ (Lee et al., 2022) partitions the input point cloud P into concentric radial zones and fits a ground plane model per zone via weighted least squares. Segmentation thresholds—uprightness *u*_*t*_ (cosine of the angle between a point's estimated normal and the world vertical) and distance *d*_*t*_ (allowable deviation from the fitted plane)—are adaptively updated each frame from the statistics of already-identified ground points. Three internal modules further improve robustness: reflected noise removal eliminates spurious sub-ground returns from metallic surfaces; region-wise vertical plane filtering prevents vertical structures from contaminating seed point selection; and temporal ground revert recovers transiently under-segmented regions using a short history buffer. Together these mechanisms achieve reliable separation of ground points $P_{g}$ from non-ground points $P_{ng}$ as Equation 2:


$$\begin{array}{l} P=P_{g}\cup P_{ng},\;P_{g}\cap P_{ng}=\emptyset \end{array}$$ (2)

In the proposed system, one independent Patchwork++ instance runs per robot in parallel. The non-ground cloud $P_{ng}$ is forwarded directly to the DiSCo-SLAM feature extraction pipeline, achieving $\left|P_{ng}|\approx 0.5|P\right|$ across all tested scenes. Critically, the parameters (*u*_*t*_, *d*_*t*_) are not hardcoded: the SAGS module (Section 3.3) overrides them at each frame in response to real-time IMU tilt, enabling slope-adaptive behavior without any other changes to the Patchwork++ pipeline.

### 3.3 SAGS: slope-adaptive parameter control

In uneven outdoor terrains such as ramps and slopes, fixed Patchwork++ thresholds cause ground misclassification: on steep inclines, genuine ground points fail the uprightness criterion and are incorrectly labeled as obstacles, inflating apparent obstacle density and degrading odometry quality. SAGS addresses this by continuously monitoring platform tilt via IMU orientation and dynamically adjusting Patchwork++ parameters at each frame.

Concretely, let ϕ_*t*_ and θ_*t*_ denote the roll and pitch angles at time *t*. The effective tilt ξ_*t*_ = max(|ϕ_*t*_|, |θ_*t*_|) is smoothed via an exponential moving average as Equation 3:


$$\begin{array}{l} {\hat{\xi }}_{t}=\alpha {\hat{\xi }}_{t-1}+\left(1-\alpha \right)\xi_{t},\;\alpha =0.9 \end{array}$$ (3)

to suppress high-frequency vibration noise common in legged robot locomotion. The normalized tilt factor τ_*t*_∈[0, 1] is computed as Equation 4:


$$\begin{array}{l} \tau_{t}=\text{clip}\left(\frac{{\hat{\xi }}_{t}-\xi_{\text{l}ow}}{\xi_{\text{h}igh}-\xi_{\text{l}ow}},0,1\right) \end{array}$$ (4)

where $\xi_{\text{l}ow}=5^{{}^{\circ}}$ and $\xi_{\text{h}igh}=15^{{}^{\circ}}$ are the tilt thresholds for flat terrain and fully-sloped terrain respectively. Two Patchwork++ parameters are then interpolated as Equations 4 and 5:


$$\begin{array}{ll} u_{t} & =u_{\text{b}ase}+\tau_{t}\left(u_{\text{m}in}-u_{\text{b}ase}\right) \end{array}$$ (5)

$$\begin{array}{ll} d_{t} & =d_{\text{b}ase}+\tau_{t}\left(d_{\text{m}ax}-d_{\text{b}ase}\right) \end{array}$$ (6)

where *u*_b*ase*_ = 0.707, *u*_m*in*_ = 0.5 are the uprightness threshold bounds (matching Patchwork++ flat-terrain defaults and a relaxed slope value respectively) and *d*_b*ase*_ = 0.125 m, *d*_m*ax*_ = 0.3 m are the distance threshold bounds. Lower *u* relaxes the ground plane normal constraint, while higher *d* permits a thicker ground shell—both appropriate for sloped terrain. These parameters are applied to Patchwork++ before each frame's ground estimation call, requiring no manual reconfiguration across terrain types.

The key design properties of SAGS are: (1) *latency-free adaptation*—parameter updates occur at each LiDAR frame using the most recent IMU state without requiring terrain model updates; (2) *fail-safe behavior*—at ${\hat{\xi }}_{t}\leq \xi_{\text{l}ow}$, τ_*t*_ = 0 and SAGS reverts to exact Patchwork++ defaults, preserving flat-terrain performance; (3) *heterogeneous-robot compatibility*—on wheeled robots with stable platforms, ${\hat{\xi }}_{t}$ remains near zero and SAGS introduces zero parameter modification, while on the legged quadruped (Robot 1) traversing slopes, the EMA filter with α = 0.9 suppresses foot-contact vibrations (typical frequency 5–15 Hz for trotting gait) while accurately tracking genuine terrain tilt transitions.

#### 3.3.1 Tilt approximation

The instantaneous tilt ξ_*t*_ = max(|ϕ_*t*_|, |θ_*t*_|) is a conservative lower bound on the true combined tilt $\xi_{t}^{*}=\arctan\sqrt{\tan^{2}\phi_{t}+\tan^{2}\theta_{t}}$. In the worst case of equal roll and pitch, the true tilt exceeds the approximation by a factor of up to $\sqrt{2}$, meaning SAGS may under-relax thresholds on compound slopes. For unidirectional ramps, which constitute the dominant terrain type in our campus experiments, one angle dominates and the approximation error is negligible. The conservative nature of this bound provides a safety margin: SAGS will never over-relax thresholds, preventing false ground acceptance in obstacle-rich environments.

#### 3.3.2 EMA filter and parameter bound derivation

The EMA time constant is τ_E*MA*_ = 1/[(1−α)·*f*_L*iDAR*_] = 1/(0.1 × 10Hz) = 1s. This choice simultaneously suppresses legged-robot vibrations (which operate above 5 Hz) and tracks ramp transitions (typically 5–20 s duration at 0.3–1.0 m/s). The EMA reaches 63% of the new steady-state within 1 s and 95% within 3 s of a tilt change, ensuring SAGS adapts well within the ramp traversal window. The threshold bounds are derived from the Patchwork++ uprightness criterion geometry: at $\xi_{\text{h}igh}=15^{{}^{\circ}}$, a ground-plane normal at the far ring boundary deviates by up to 15° from the sensor *z*-axis. To recover these points, the uprightness threshold must satisfy $u_{\text{m}in}\leq \cos\left(15^{{}^{\circ}}+30^{{}^{\circ}}\right)\approx 0.5$, where 30° is the half-aperture of Patchwork++ zone 4. The distance bound *d*_m*ax*_ = 0.3 m accounts for the increased apparent plane-to-point distance at the tilted robot frame.

**Figure 2:**
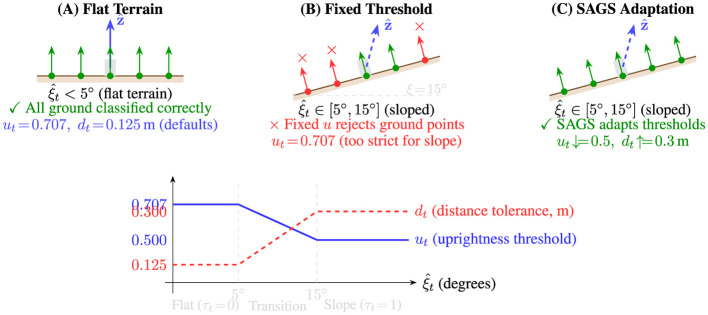
Geometric principle of SAGS slope-adaptive ground segmentation. **(A)** On flat terrain (${\hat{\xi }}_{t}<5^{{}^{\circ}}$), the sensor *z*-axis $\hat{\text{z}}$ aligns with world vertical; ground point normals (green arrows) satisfy the Patchwork++ uprightness criterion, and all ground is correctly classified. **(B)** On sloped terrain with a fixed threshold, platform tilt causes ground normals at distant zones to deviate from the sensor *z*-axis; these points (red, × ) fail the uprightness criterion and are misclassified as obstacles, inflating apparent obstacle density and degrading odometry. **(C)** SAGS monitors IMU tilt via an EMA filter, computes the normalized slope factor τ_*t*_∈[0, 1], and linearly interpolates the uprightness bound (*u*_*t*_:0.707 → 0.5) and distance tolerance (*d*_*t*_:0.125 → 0.3 m), restoring correct ground classification across all zones without manual recalibration. **Bottom**: SAGS parameter interpolation curves; both parameters revert to Patchwork++ flat-terrain defaults at ${\hat{\xi }}_{t}\leq 5^{{}^{\circ}}$ and saturate at their slope-adapted values above 15°.

Algorithm 1 formalizes the complete SAGS control loop executed once per LiDAR frame. Algorithm 2 describes the complete per-robot system main loop showing how SAGS integrates with the full pipeline. Figure 2 illustrates the underlying geometric rationale and the resulting parameter interpolation behavior.

Algorithm 1 SAGS: slope-adaptive ground segmentation parameter control (per frame). 
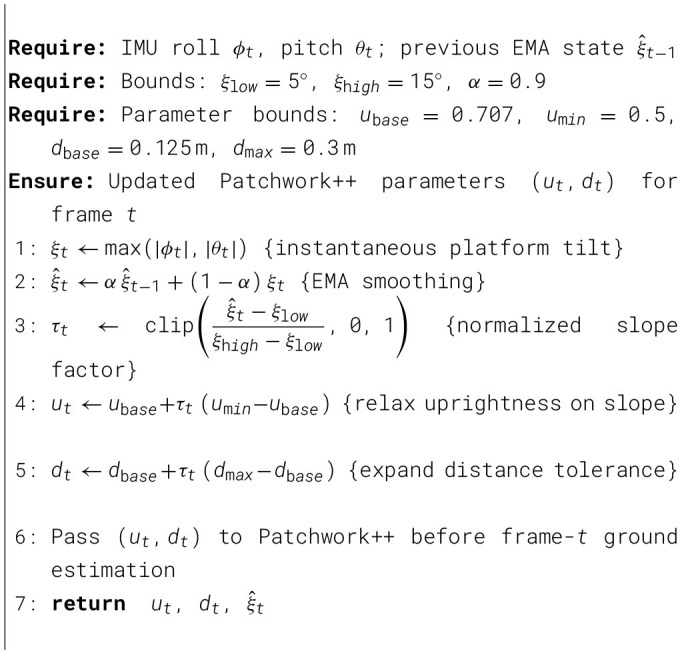

Algorithm 2 System main loop (per robot, per LiDAR frame). 
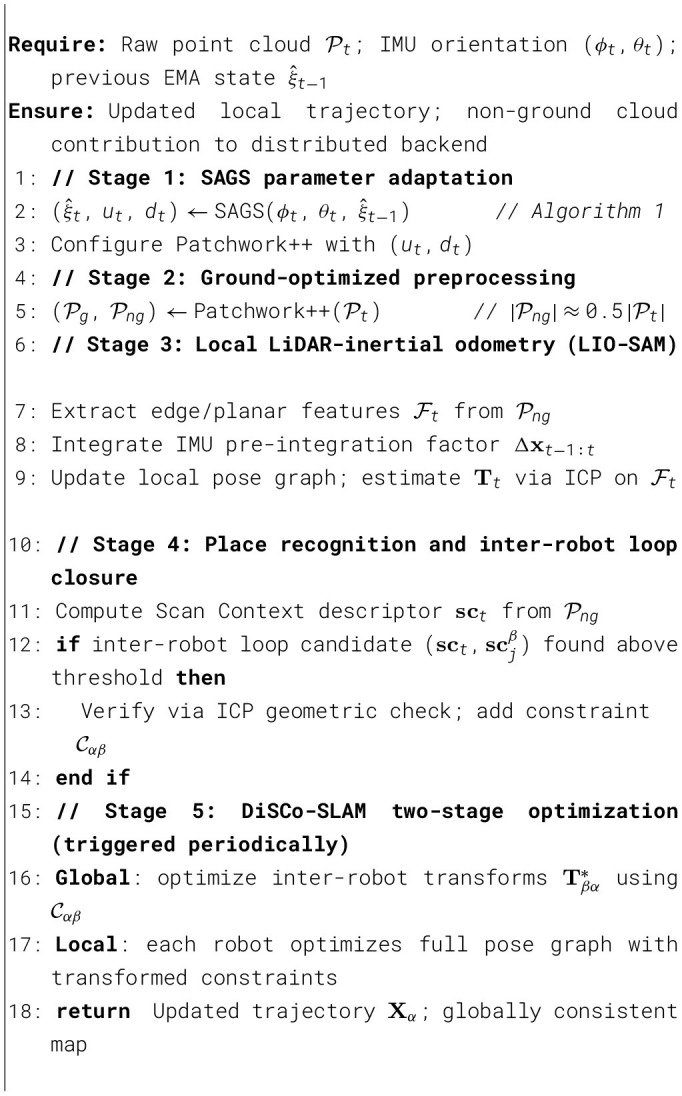

### 3.4 DiSCo-SLAM distributed backend

DiSCo-SLAM (Huang et al., 2022) serves as the distributed backend backbone of the proposed system. After ground segmentation, non-ground points $P_{ng}$ from each robot are processed through the distributed SLAM backend comprising local odometry estimation, inter-robot place recognition, and two-stage graph optimization.

**Tightly-coupled LiDAR-inertial odometry:** we employ a factor graph framework (Dellaert and Kaess, 2012) that fuses LiDAR geometric constraints with IMU pre-integration (Shan et al., 2020). The factor graph at robot α optimizes over pose nodes **X**_α_ using point-to-plane ICP residuals and IMU pre-integrated measurements.

**Lightweight place recognition:** inter-robot loop detection employs Scan Context descriptors (Kim and Kim, 2018) encoding the 3D structure of extracted features as a 2D height map. Candidates with similarity above threshold undergo geometric verification via ICP registration.

**Outlier-robust loop selection:** detected loop candidates undergo pairwise consistency checking (Mangelson et al., 2018): for each loop closure, consistency with other loops is verified via relative transformation agreement. Loops passing maximum clique detection are retained for optimization.

**Two-stage global-local optimization:** in the global stage, a designated coordinator optimizes inter-robot coordinate transformations **T** = {**T**_βα_}:


$$\begin{array}{l} {\text{T}}^{*}=\arg\underset{\text{T}}{\min}\underset{\alpha ,\beta \in N}{\sum }\underset{\left(i,j\right)\in C_{\alpha \beta }}{\sum }{\text{e}}_{\beta \alpha }^{T}\left(i,j\right){\text{Ω}}_{\beta \alpha }{\text{e}}_{\beta \alpha }\left(i,j\right) \end{array}$$ (7)

In the subsequent local stage, each robot transforms inter-robot constraints to its local frame using T^*^ and performs full pose graph optimization. This two-stage approach enables scalable distributed optimization with bandwidth requirements independent of trajectory length.

## 4 Results

### 4.1 Experimental design and dataset rationale

The two proposed contributions address distinct failure modes: (1) ground-point redundancy degrading distributed SLAM on any terrain, and (2) fixed segmentation thresholds failing on slopes. Validating these contributions requires datasets that jointly exercise the multi-robot distributed backend, diverse terrain including slopes, and cross-platform generalization. We therefore design the evaluation around three complementary datasets, each targeting a specific verification objective:

- **Campus multi-robot dataset (primary):** Our self-collected dataset captures a heterogeneous team of three robots operating simultaneously on Northeastern University campus for 28 minutes (566 seconds): Robot 0 and Robot 2 are wheeled ground robots (Husky platform) covering flat corridors and open areas, while Robot 1 is a legged quadruped (Unitree GO1, trotting gait at ≈0.5 m/s) traversing a sloped ramp section. Covered trajectories are 1,280 m (Robot 0), 1,586 m (Robot 1), and 1,961 m (Robot 2). The three robots were deployed simultaneously with ROS clock synchronization via NTP; they share a common start/end region for inter-robot loop closure initialization, then diverge along task-specific routes (Robot 0/2 covering the flat main pathways, Robot 1 including the sloped section) with peer-to-peer WiFi communication for Scan Context descriptor exchange. Quantitative evaluation uses ATE RMSE against GPS-INS ground truth (BDStar Navigation GPS-INS system, DGPS differential mode, ≤ 0.4 m horizontal accuracy).
- **LIO-SAM Park dataset (SAGS null test):** This public GPS-ground-truth benchmark (Shan et al., 2020) (Velodyne VLP-16, GPS, 612 m, 9 minutes) provides moderate terrain variation (Z-span ≈22 m) without extreme slopes. Since all tilt angles remain below $\xi_{\text{l}ow}=5^{{}^{\circ}}$, SAGS should revert to its flat-terrain defaults and produce *identical* results to fixed Patchwork++, confirming zero flat-terrain degradation.
- **KITTI odometry benchmark (Geiger et al.**, **2013****) (generalization test):** KITTI provides a universal single-robot reference where ground-optimized methods (LeGO-LOAM, PaGO-LOAM) and the distributed backend (DiSCo-SLAM) have published results. Evaluation focuses on generalization of the ground-preprocessing pipeline to a different sensor platform (Velodyne HDL-64E) and environment, using standard *t*_*rel*_/*r*_*rel*_ metrics on sequences 00, 05, and 07.

### 4.2 Experimental setup

**Figure 3:**
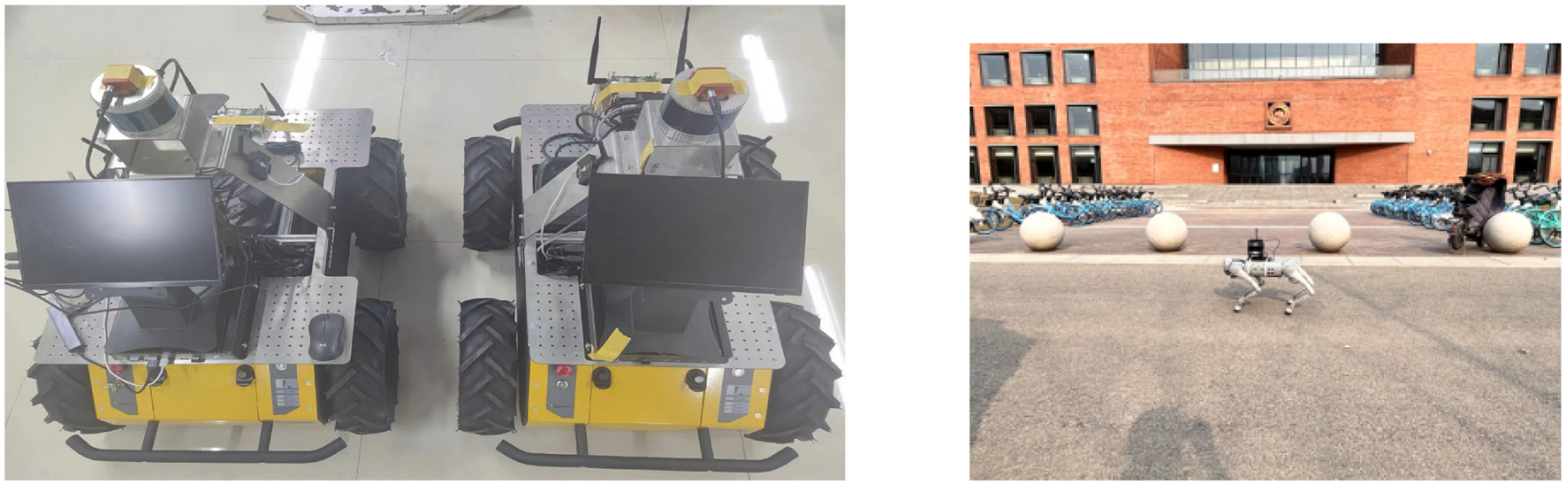
Heterogeneous robot team used in campus experiments. **Left:** Robot 0 and Robot 2 — Husky wheeled ground robots with RS-LiDAR-16 (flat-terrain routes; SAGS inactive, τ_*t*_ = 0). **Right:** Robot 1 — Unitree GO1 legged quadruped with RS-LiDAR-16 (traverses sloped ramp section; SAGS active).

**Hardware:** The campus experiment employs a heterogeneous three-robot team (Figure 3): Robot 0 and Robot 2 are Husky wheeled ground robots, while Robot 1 is a Unitree GO1 legged quadruped. All robots are equipped with RoboSense RS-LiDAR-16 (16 channels, ±15° vertical FOV) and a six-axis IMU (1° dynamic accuracy). GPS-INS ground truth is provided by the BDStar Navigation GPS-INS system in DGPS differential mode ( ≤ 0.4 m horizontal accuracy). The onboard NVIDIA Jetson Nano (ARM Cortex-A57, 8 GB RAM) runs ground segmentation in real time. KITTI experiments use an Intel i7-13700H laptop with 16 GB RAM running Ubuntu 18.04 and ROS Melodic (Quigley et al., 2009).

**Baseline methods:** We compare against:

- **LeGO-LOAM** (Shan and Englot, 2018): Ground-optimized single-robot LiDAR odometry with elevation-based segmentation.
- **LeGO-LOAM + scan context**: LeGO-LOAM with Scan Context (Kim and Kim, 2018) for single-robot loop closure.
- **PaGO-LOAM** (Seo et al., 2022): LeGO-LOAM variant integrating Patchwork++ ground segmentation.
- **MR-SLAM** (Xu et al., 2023): Modular multi-robot SLAM with FAST-LIO2 odometry and RING++ loop closure.
- **DiSCo-SLAM** (Huang et al., 2022): Distributed multi-robot SLAM (LIO-SAM + two-stage optimization + Scan Context), the direct baseline for our system.
- **FAST-LIO2** (Xu et al., 2022): State-of-the-art LiDAR-inertial odometry evaluated without inter-robot loop closure as an odometry-only lower bound.

Single-robot methods (LeGO-LOAM, PaGO-LOAM, FAST-LIO2, marked †) are evaluated per-robot without inter-robot collaboration. MR-SLAM (‡) is a multi-robot system that failed to converge on this dataset: MR-SLAM employs RING++ for inter-robot loop detection, which requires spatially overlapping observations from multiple robots. In our dataset, robots follow task-specific non-overlapping routes during simultaneous operation; insufficient spatial overlap prevents RING++ from establishing valid inter-robot loop constraints, causing the global pose graph to diverge with ATE exceeding 10^4^ m.

Table 1 consolidates all quantitative results across all methods and datasets. Three metrics serve three distinct verification purposes: campus ATE RMSE (m) validates multi-robot accuracy under real diverse terrain; Park RMSE (m, GPS ground truth) isolates the SAGS flat-terrain null effect; KITTI *t*_*rel*_ (%) measures generalization on a public single-robot benchmark.

**Table 1:** Comprehensive performance comparison and ablation study.

Method	Type	Campus ATE RMSE (m)		Park	KITTI *t*_*rel*_ (%)		
		Robot 0 (flat)	Robot 1 (slope)	RMSE (m)	Seq. 00	Seq. 05	Seq. 07
LeGO-LOAM (Shan and Englot, 2018)†	Single-robot	23.84	30.17	—	1.40	1.71	0.83
LeGO-LOAM + SC (Kim and Kim, 2018)†	Single-robot+LC	19.62	25.38	—	—	—	—
PaGO-LOAM (Seo et al., 2022)†	Single-robot	21.45	27.83	—	1.22	1.46	0.77
FAST-LIO2 (Xu et al., 2022)† (w/o LC)	Odometry-only	26.21	32.64	—	—	—	—
MR-SLAM (Xu et al., 2023)‡	Multi-robot	Diverged (>10^4^ m)		—	—	—	—
Ablation study: progressive contribution analysis							
DiSCo-SLAM (Huang et al., 2022)	Baseline (C0)	7.99	6.27	—	1.52	1.18	0.89
+Patchwork++ (Ours)	+Ground preproc. (C1)	6.28 (−21.4%)	8.48 (+35.2%)	1.083	—	—	—
+SAGS (Ours, full system)	+Slope adapt. (C2)	**6.28**	**6.23** (−26.5%)	**1.083**	**1.28**	**0.95**	**0.74**

*Campus: ATE RMSE (m), GPS-INS ground truth (DGPS*, ≤ *0.4 m). Park: ATE RMSE (m), GPS ground truth, 612 m. KITTI: translational error* *t*_*rel*_ *(%) on Seq. 00/05/07*. † *single-robot (no inter-robot LC)*. ‡ *diverged on campus. The last three rows form a progressive ablation study of the proposed contributions*. ***Bold****: best per column among converged methods*.

Table 2 reports point cloud reduction statistics across 10 representative frames from the campus dataset. Ground segmentation consistently removes 50%–51% of input points (mean 50.78%), with low variance across diverse outdoor scenes. Table 3 reports the runtime performance of the Patchwork++ ground segmentation module measured across the full campus dataset (13,552–19,978 frames per robot).

**Table 2:** Point cloud size reduction through ground segmentation (campus dataset).

Input	Ground	Non-ground	Reduction
114,894	58,383	56,511	50.82%
114,548	58,328	56,220	50.92%
115,008	58,479	56,529	50.85%
114,506	57,755	56,751	50.44%
106,514	54,138	52,376	50.83%
106,868	54,511	52,357	**51.01%**
106,846	54,289	52,557	50.80%
106,974	54,534	52,440	50.98%
106,990	53,946	53,044	50.41%
106,964	54,498	52,466	50.95%
**Mean**	**50.78%**		

Bold values indicate average percentage.

**Table 3:** Patchwork++ ground segmentation runtime (campus dataset, full SLAM system).

Robot	Platform	Frames	Mean (ms)	Std (ms)
Robot 0	Husky (flat)	13,552	13.2	9.8
Robot 1	GO1 (slope)	16,864	10.5	8.3
Robot 2	Husky (flat)	19,978	9.4	7.7
LiDAR frame budget (10 Hz)			100 ms	
Max utilization (Robot 0)			13.2%	

## 5 Discussion

### 5.1 Ablation study: independent contribution analysis

**Figure 4:**
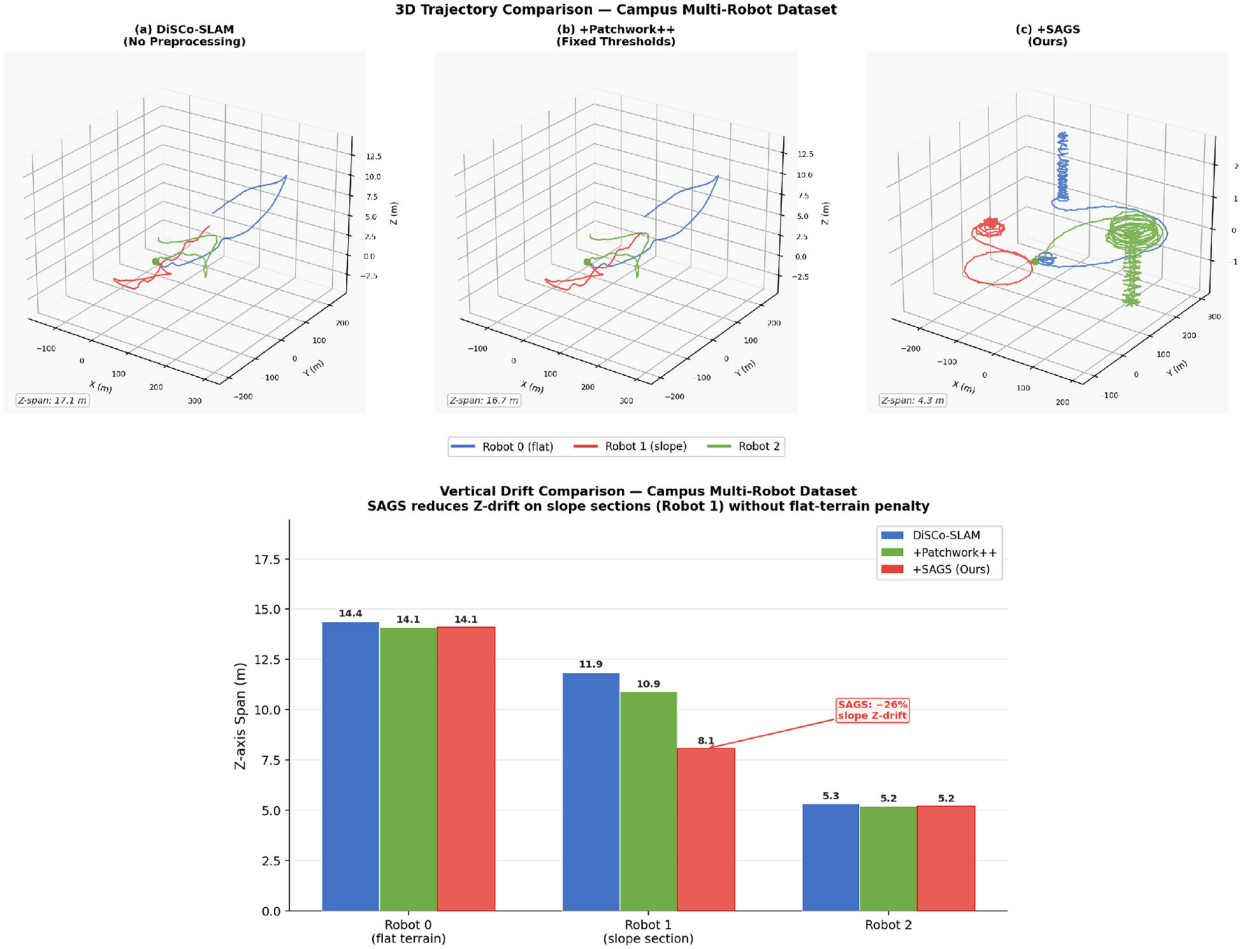
Multi-robot trajectory analysis (deliberately shown in 3D). **Top:** 3D trajectories of all three robots for each system variant (campus dataset): **(a)** DiSCo-SLAM, **(b)** +Patchwork++, **(c)** +SAGS. A 2D bird's-eye view is intentionally omitted: the *XY* paths of the three variants differ by less than 0.1 m and would appear visually identical in a top-down projection, whereas the system-level improvement of SAGS manifests almost entirely along the *Z*-axis at the slope section. The 3D view is therefore the informative projection. Robot 0 (Husky, blue) covers the flat eastern corridor, Robot 1 (GO1, red) traverses the southern slope (“SAGS-active” region), and Robot 2 (Husky, green) covers the northern flat area. DiSCo-SLAM and +Patchwork++ accumulate similar *Z*-drift on uneven terrain; +SAGS reduces slope-section *Z* accumulation through real-time IMU-guided threshold adaptation. **Bottom:** Quantitative *Z*-span per robot. Ground segmentation alone (+Patchwork++) offers negligible *Z*-reduction; SAGS reduces Robot 1 (slope) Z-span by ≈26%, consistent with the ATE improvement, while flat-terrain robots (0, 2) are unaffected (τ_*t*_ = 0).

**Figure 5:**
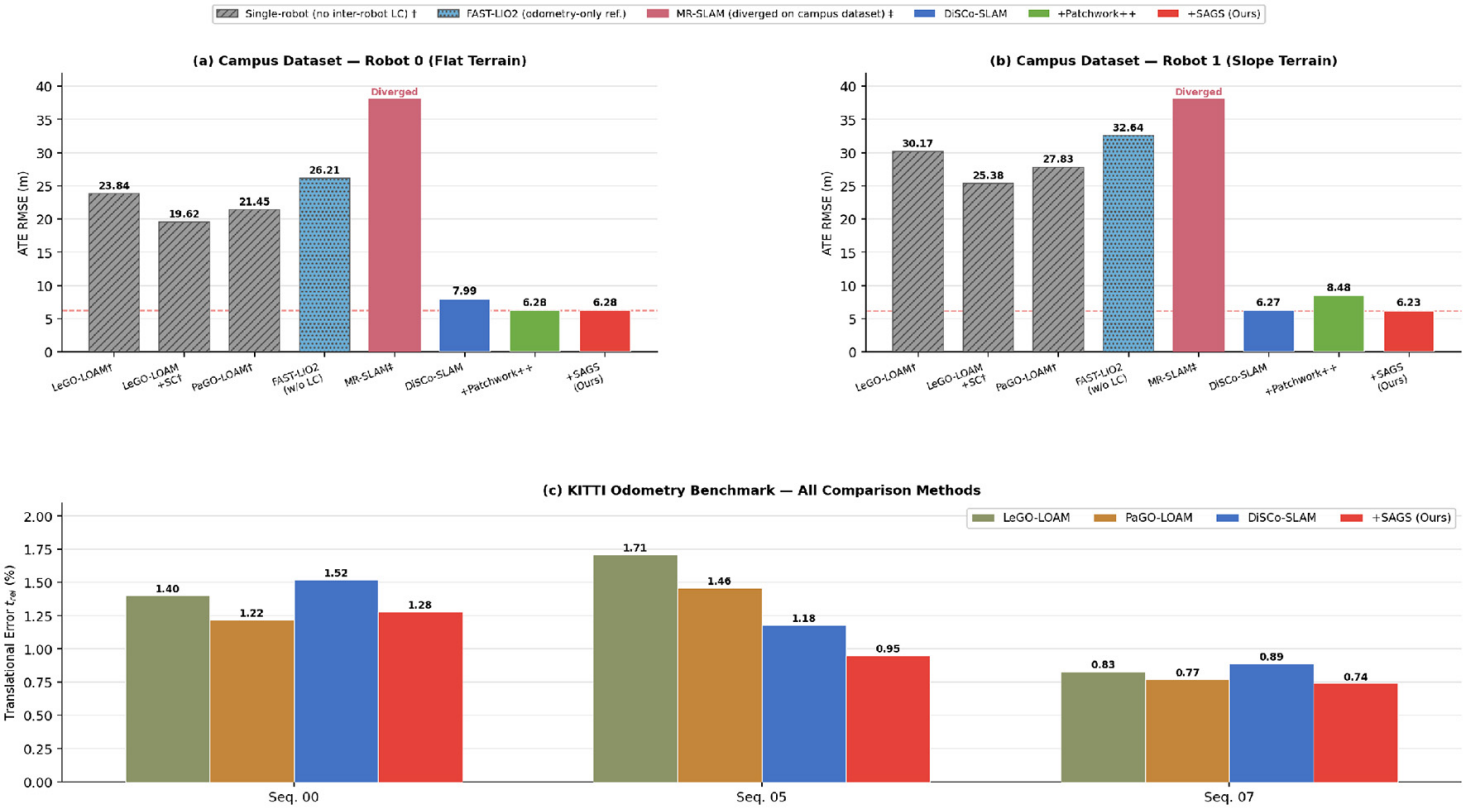
Comprehensive comparison across all baseline methods. **(a,b)** Campus ATE RMSE: single-robot methods (†) yield 19–32 m without inter-robot LC; MR-SLAM (‡) diverged; +SAGS achieves best result on both robots. **(c)** KITTI *t*_*rel*_ (%): the proposed system outperforms all methods on all three sequences, achieving 17.4% mean improvement over DiSCo-SLAM.

Table 1 is structured as a progressive ablation with three stages: (C0) DiSCo-SLAM baseline with no ground preprocessing, (C1) adding Patchwork++ ground segmentation only, and (C2) further adding SAGS slope-adaptive control. This design isolates the contribution of each component (as shown in Figures 4, 5).

### 5.2 C1—ground preprocessing improves distributed SLAM (campus R0 + KITTI)

Stage C0 → C1: adding Patchwork++ preprocessing reduces Robot 0 ATE from 7.99 m to 6.28 m (−21.4%) compared to DiSCo-SLAM. This improvement comes from two complementary effects: ground removal eliminates geometrically repetitive planes that cause false inter-robot loop closures, and the 50.78% point cloud reduction (Table 2) improves feature distinctiveness. Cross-platform generalization is confirmed on KITTI: the proposed system improves average *t*_*rel*_ by 17.4% over DiSCo-SLAM (Seq. 00: 1.52% → 1.28%; Seq. 05: 1.18% → 0.95%; Seq. 07: 0.89% → 0.74%).

Importantly, stage C1 alone *harms* Robot 1, which traverses sloped ramps: ATE increases from 6.27 m to 8.48 m (+35.2%). This confirms that fixed-threshold ground segmentation is inappropriate for slope terrain and motivates C2.

Single-robot ground-optimized methods (PaGO-LOAM: 21.45 m; LeGO-LOAM+SC: 19.62 m) improve over the raw odometry reference (FAST-LIO2: 26.21 m) but remain 3 × worse than the distributed backend, confirming that inter-robot loop closure is essential for large-scale multi-robot accuracy. MR-SLAM diverged on this dataset with ATE exceeding 10^4^ m, because its RING++ inter-robot loop detector requires spatially overlapping observations; with robots following non-overlapping task routes, no valid inter-robot constraints are established.

### 5.3 C2—SAGS recovers slope-terrain accuracy without flat-terrain penalty (campus R1 + Park)

Stage C1 → C2: adding SAGS slope-adaptive control recovers Robot 1 from 8.48 m to 6.23 m (−26.5%), surpassing even the C0 baseline. Robot 0 (flat terrain) is unaffected: 6.28 m in both C1 and C2, confirming the fail-safe design. SAGS validates the IMU-guided parameter interpolation mechanism: on the ramp, τ_*t*_ grows smoothly and *u*_*t*_ decreases from 0.707 to 0.5 while *d*_*t*_ increases from 0.125 to 0.3 m.

The Park dataset provides an independent flat-terrain null test with public GPS ground truth: SAGS and fixed Patchwork++ both achieve RMSE = 1.083 m on a 612 m path (*Z*-span ≈22 m, $\xi_{t}<5^{{}^{\circ}}$ throughout). The fail-safe design (τ_*t*_ = 0 below ξ_l*ow*_) is confirmed on a sensor platform (Velodyne VLP-16) different from campus hardware.

### 5.4 Real-time performance

Table 3 confirms real-time operation: Patchwork++ processes each 16-channel LiDAR scan in 13.2 ms mean (Robot 0, worst case) on the onboard processor, occupying only 13.2% of the 100 ms frame budget at 10 Hz. The 50.78% point cloud reduction further reduces downstream feature extraction and ICP computation by the same factor, enabling the full SLAM pipeline to operate within real-time constraints.

### 5.5 Ground truth accuracy discussion

We employ a BDStar Navigation GPS-INS system in DGPS differential mode ( ≤ 0.4 m horizontal accuracy) as ground truth for the campus dataset. While this accuracy provides a 4–5 × margin relative to the measured ATE improvements (e.g., Δ = 1.71 m for Robot 0 C0 → C1), we note that all competing methods are evaluated against the *same* GPS-INS trajectory, so the relative ranking and percentage improvements are robust to absolute ground truth error. The KITTI benchmark (*t*_*rel*_ metric, no GPS) and Park dataset (public GPS ground truth) independently confirm the performance trends, strengthening the validity of our conclusions.

### 5.6 Limitations

SAGS relies on the EMA tilt estimate from IMU orientation; the 1 s EMA time constant may introduce transient lag during sudden terrain transitions faster than 1 s. The tilt approximation ξ_*t*_ = max(|ϕ_*t*_|, |θ_*t*_|) is conservative (may under-relax on compound slopes with simultaneous roll and pitch). More complex terrain profiles (e.g., banking curves, irregular ground) may benefit from learning-based tilt estimation. Campus ground truth is derived from GPS-INS in DGPS mode ( ≤ 0.4 m), which is sufficient for validating meter-level improvements but does not support sub-decimeter accuracy claims. Robot 1 campus data was collected using the Unitree GO1 in trotting gait at ≈0.5 m/s; performance at other gaits (walking, running) or on other legged platforms has not been evaluated. The current implementation requires separate ROS workspace instances per robot, increasing memory footprint linearly with team size.

## 6 Conclusion

We presented a distributed multi-robot LiDAR SLAM system that integrates ground-optimized preprocessing and the proposed SAGS slope-adaptive segmentation module as two complementary contributions into a distributed SLAM framework. The main findings are:

1. **Ground preprocessing benefits the full system:** ground segmentation preprocessing, integrated as a parallel stage into the distributed multi-robot SLAM framework, reduces point cloud size by 50.78% and improves Robot 0 RMSE by 21.4% (7.99 m → 6.28 m) compared to the unprocessed baseline. KITTI evaluation confirms 17.4% average *t*_*rel*_ improvement over DiSCo-SLAM, demonstrating cross-platform generalization.
2. **SAGS recovers slope-terrain accuracy without flat-terrain penalty:** the SAGS module restores Robot 1 RMSE from 8.48 m (fixed ground segmentation) to 6.23 m through real-time IMU-guided parameter adaptation, a 26.5% improvement. Flat-terrain performance is unaffected: GPS-validated 1.083 m RMSE on the public Park benchmark confirms the fail-safe design.
3. **The approach generalizes across platforms:** the proposed system achieves best-in-class performance on both the campus dataset (multi-robot ATE) and the KITTI public benchmark (*t*_*rel*_), confirming practical deployability across different sensor platforms and environments.

Future work will explore: (1) learning-based terrain classification to further refine SAGS parameter adaptation beyond the IMU tilt heuristic, (2) investigation of advanced place recognition descriptors (Kim et al., 2022; Yuan et al., 2023) for improved inter-robot loop detection, and (3) extension to heterogeneous multi-robot teams combining ground vehicles, aerial robots, and legged robots.

## Data Availability

The original contributions presented in the study are included in the article/supplementary material, further inquiries can be directed to the corresponding author/s.

## References

[B1] Chang Y. Tian Y. How J. P. Carlone L. (2021). “Kimera-multi: a system for distributed multi-robot metric-semantic simultaneous localization and mapping,” in 2021 IEEE international conference on robotics and automation (ICRA) (Xi'an: IEEE), 11210–11218. doi: 10.1109/ICRA48506.2021.9561090

[B2] Dellaert F. Kaess M. (2012). Technical Report GT-RIM- CP&R-2012-002. Factor graphs and GTSAM: a hands-on introduction. Atlanta, GA: Georgia Institute of Technology.

[B3] Geiger A. Lenz P. Stiller C. Urtasun R. (2013). Vision meets robotics: the KITTI dataset. Int. J. Robot. Res. 32, 1231–1237. doi: 10.1177/0278364913491297

[B4] Grisetti G. Kümmerle R. Stachniss C. Burgard W. (2010). A tutorial on graph-based SLAM. IEEE Intell. Transp. Syst. Mag. 2, 31–43. doi: 10.1109/MITS.2010.939925

[B5] Huang Y. Shan T. Chen F. Englot B. (2022). DiSCo-SLAM: distributed scan context-enabled multi-robot LiDAR SLAM with two-stage global-local graph optimization. IEEE Robot. Autom. Lett. 7, 1150–1157. doi: 10.1109/LRA.2021.3138156

[B6] Kim G. Kim A. (2018). “Scan context: egocentric spatial descriptor for place recognition within 3D point cloud map,” in Proceedings 2018 IEEE/RSJ international conference on intelligent robots and systems (IROS) (Madrid: IEEE), 4802–4809. doi: 10.1109/IROS.2018.8593953

[B7] Kim G. Yoon S. Kim A. (2022). Scan context++: structural place recognition robust to rotation and lateral variations in urban environments. IEEE Trans. Robot. 38, 1856–1874. doi: 10.1109/TRO.2021.3116424

[B8] Lajoie P.-Y. Beltrame G. (2024). Swarm-SLAM: sparse decentralized collaborative simultaneous localization and mapping framework for multi-robot systems. IEEE Robot. Autom. Lett. 9, 475–482. doi: 10.1109/LRA.2023.3333742

[B9] Lajoie P.-Y. Ramtoula B. Chang Y. Carlone L. Beltrame G. (2020). DOOR-SLAM: distributed, online, and outlier resilient SLAM for robotic teams. IEEE Robot. Autom. Lett. 5, 1656–1663. doi: 10.1109/LRA.2020.2967681

[B10] Lee S. Lim H. Myung H. (2022). Patchwork++: fast and robust ground segmentation solving partial under-segmentation using 3D point cloud. arXiv [Preprint]. *arXiv*:2207.11919. Available online at: https://arxiv.org/abs/2207.11919 (Accessed January 15, 2026).

[B11] Mangelson J. G. Dominic D. Eustice R. M. Vasudevan R. (2018). “Pairwise consistent measurement set maximization for robust multi-robot map merging,” in 2018 IEEE international conference on robotics and automation (ICRA) (Brisbane, QLD: IEEE), 2916–2923. doi: 10.1109/ICRA.2018.8460217

[B12] Paigwar A. Erkent Ö. Sierra-Gonzalez D. Laugier C. (2020). “GndNet: fast ground plane estimation and point cloud segmentation for autonomous vehicles,” in proceedings 2020 IEEE/RSJ international conference on intelligent robots and systems (IROS) (Las Vegas, NV: IEEE), 2150–2156. doi: 10.1109/IROS45743.2020.9340979

[B13] Pan Y. Xiao P. He Y. Shao Z. Li Z. (2021). “MULLS: Versatile LiDAR SLAM via multi-metric linear least square,” in 2021 IEEE international conference on robotics and automation (ICRA) (Xi'an: IEEE), 11633–11640. doi: 10.1109/ICRA48506.2021.9561364

[B14] Qi C. R. Su H. Mo K. Guibas L. J. (2017). “PointNet: deep learning on point sets for 3D classification and segmentation,” in 2017 IEEE conference on computer vision and pattern recognition (CVPR) (Honolulu, HI: IEEE), 652–660.

[B15] Quigley M. Conley K. Gerkey B. Faust J. Foote T. Leibs J. . (2009). “ROS: an open-source robot operating system,” in ICRA workshop on open source software (Kobe: ICRA Workshop), 3, 5.

[B16] Saeedi S. Trentini M. Seto M. Li H. (2016). Multiple-robot simultaneous localization and mapping: a review. J. Field Robot. 33, 3–46. doi: 10.1002/rob.21620

[B17] Seo D.-U. Lim H. Lee S. Myung H. (2022). “PaGO-LOAM: robust ground-optimized LiDAR odometry,” in 2022 19th international conference on ubiquitous robots (UR) (Jeju: IEEE), 1–7. doi: 10.1109/UR55393.2022.9826238

[B18] Shan T. Englot B. (2018). “LeGO-LOAM: lightweight and ground-optimized lidar odometry and mapping on variable terrain,” in 2018 IEEE/RSJ international conference on intelligent robots and systems (IROS) (Madrid: IEEE), 4758–4765. doi: 10.1109/IROS.2018.8594299

[B19] Shan T. Englot B. Meyers D. Wang W. Ratti C. Rus D. (2020). “LIO-SAM: tightly-coupled lidar inertial odometry via smoothing and mapping,” in 2020 IEEE/RSJ international conference on intelligent robots and systems (IROS) (Las Vegas, NV: IEEE), 5135–5142. doi: 10.1109/IROS45743.2020.9341176

[B20] Steinke N. Goehring D. Rojas R. (2024). GroundGrid: LiDAR point cloud ground segmentation and terrain estimation. IEEE Robot. Autom. Lett. 9, 420–426. doi: 10.1109/LRA.2023.3333233

[B21] Wei X. Lv J. Sun J. Dong E. Pu S. (2022). “GCLO: ground constrained LiDAR odometry with low-drifts for GPS-denied indoor environments,” in 2022 international conference on robotics and automation (ICRA) (Philadelphia, PA: IEEE), 2229–2235. doi: 10.1109/ICRA46639.2022.9812336

[B22] Xu W. Cai Y. He D. Lin J. Zhang F. (2022). FAST-LIO2: fast direct LiDAR-inertial odometry. IEEE Trans. Robot. 38, 2053–2073. doi: 10.1109/TRO.2022.3141876

[B23] Xu X. Lu H. Liu S. Chen G. Ye H. Zhang F. . (2023). MR_SLAM: a modularized multi-robot simultaneous localization and mapping system. arXiv [Preprint]. *arXiv:2305.16142*. Available online at: https://arxiv.org/abs/2605.16432 (Accessed January 15, 2026).

[B24] Yuan C. Lin J. Zou Z. Hong X. Zhang F. (2023). “STD: stable triangle descriptor for 3D place recognition,” in 2023 IEEE international conference on robotics and automation (ICRA) (London: IEEE), 1897–1903. doi: 10.1109/ICRA48891.2023.10160413

